# Different Evolutionary Origins for the Reach and the Grasp: An Explanation for Dual Visuomotor Channels in Primate Parietofrontal Cortex

**DOI:** 10.3389/fneur.2013.00208

**Published:** 2013-12-23

**Authors:** Jenni M. Karl, Ian Q. Whishaw

**Affiliations:** ^1^Department of Neuroscience, Canadian Centre for Behavioural Neuroscience, University of Lethbridge, Lethbridge, AB, Canada

**Keywords:** prehension, Reach, Grasp, Jeannerod, dual visuomotor channels, parietofrontal cortex, visually guided grasping, haptically guided grasping

## Abstract

The Dual Visuomotor Channel Theory proposes that manual prehension consists of two temporally integrated movements, each subserved by distinct visuomotor pathways in occipitoparietofrontal cortex. The Reach is mediated by a dorsomedial pathway and transports the hand in relation to the target’s extrinsic properties (i.e., location and orientation). The Grasp is mediated by a dorsolateral pathway and opens, preshapes, and closes the hand in relation to the target’s intrinsic properties (i.e., size and shape). Here, neuropsychological, developmental, and comparative evidence is reviewed to show that the Reach and the Grasp have different evolutionary origins. First, the removal or degradation of vision causes prehension to decompose into its constituent Reach and Grasp components, which are then executed in sequence or isolation. Similar decomposition occurs in optic ataxic patients following cortical injury to the Reach and the Grasp pathways and after corticospinal tract lesions in non-human primates. Second, early non-visual PreReach and PreGrasp movements develop into mature Reach and Grasp movements but are only integrated under visual control after a prolonged developmental period. Third, comparative studies reveal many similarities between stepping movements and the Reach and between food handling movements and the Grasp, suggesting that the Reach and the Grasp are derived from different evolutionary antecedents. The evidence is discussed in relation to the ideas that dual visuomotor channels in primate parietofrontal cortex emerged as a result of distinct evolutionary origins for the Reach and the Grasp; that foveated vision in primates serves to integrate the Reach and the Grasp into a single prehensile act; and, that flexible recombination of discrete Reach and Grasp movements under various forms of sensory and cognitive control can produce adaptive behavior.

## Introduction

Prehension, the act of reaching to grasp an object, is used for many everyday functions, the most common of which is to retrieve a food item and place it in the mouth for eating. Prehension is performed with little conscious effort and appears as a seamless act. Thus, it is not surprising that it is sometimes considered a single movement in experimental research (1–4) or that it is proposed to have a single evolutionary origin, possibly derived from walking (5), climbing through tree branches (6, 7), digging (8), or capturing prey (9).

Nonetheless, distinctive changes in prehension have been reported after brain injury as some patients display curious impairments in hand preshaping for grasping despite being able to accurately transport the hand to the location of a visual target. To explain this phenomena Jeannerod (10) proposes that prehension actually consists of two distinct but temporally integrated movements, a Reach and a Grasp, each mediated by different neural pathways which project from visual to motor cortex via the parietal lobe. The Dual Visuomotor Channel Theory (10, 11) has since received support from electrophysiological, neuroanatomical, and brain imaging studies while also generating insight into the biomechanics of prehension [for reviews see Ref. (12–15)]. Nonetheless, it does raise new questions concerning the evolutionary origins of prehension. Specifically, how did the Reach and the Grasp come to be mediated by different neural substrates? Indeed, the theory seems to suggest that prehension has not one, but two, evolutionary origins.

Various animal species display a wide range of Reach and Grasp specializations using the tongue, mouth, neck, tail, trunk, or hand, each of which can be guided by various sensory modalities including olfaction, audition, somatosensation, and vision (16, 17). Thus, evolutionary pressures favoring either the Reach or the Grasp could explain differences in forelimb specialization in different phylogenetic lineages. As an extreme example, the third digit is specialized for stepping in the horse (18) and specialized for foraging and prey capture in the aye-aye (19, 20). In primates, the Reach and the Grasp appear to have co-evolved and are put to integrated use in the many movements that comprise prehension. Nevertheless, distinct functional, biomechanical, and neuroanatomical features of the Reach and the Grasp suggest that each has its own evolutionary history.

This review re-examines the origins of primate prehension with the aim of identifying evolutionary antecedents for the Reach and the Grasp. The Dual Visuomotor Channel Theory is described first, followed by behavioral, neuropsychological, and developmental evidence that without vision prehension decomposes into discrete Reach and Grasp components. Comparative evidence is then presented to show that Reach and Grasp movements are not only identifiable in the forelimb movements of primates, but also in many non-primate species. Collectively, the evidence suggests that the Reach and the Grasp are derived from different evolutionary origins and were only recently, in phylogenetic terms, integrated together under visual control in primates.

## The Dual Visuomotor Channel Theory

The Dual Visuomotor Channel Theory has its origins in the proposal that pointing has two phases. A ballistic movement brings the forelimb to the general location of a target and then a visually guided corrective movement positions the hand on the target (21). Indeed, dual phase guidance may be a general feature of animal movement (22). The distinctive contribution of the Dual Visuomotor Channel Theory is that it describes prehension in ethological terms: the Reach serves to bring the hand into contact with the target by transporting it to the appropriate location whereas the Grasp serves to shape the hand for target purchase. As distinct behaviors, the Reach and the Grasp may be subject to different evolutionary pressures and adaptive specializations that can be analyzed by comparative methods.

Distinctive features of the Reach and the Grasp are summarized in Table 1. The Reach transports the hand to the location of the target so that the digits align with appropriate contact points on the target. It is produced largely by proximal musculature of the upper arm, is guided by the extrinsic properties of the target (location and orientation), and is coded in egocentric coordinates relative to the reacher. The Grasp preshapes the digits by first opening them to a peak aperture that scales to target size, then gradually closes them on approach to the target, and finally closes them completely for target purchase. The Grasp is produced mainly by distal musculature of the hand and digits, is guided by the intrinsic properties of the target (size and shape), and can be coded in spatial coordinates intrinsic to the hand irrespective of the hand’s location relative to the body (23).

**Table 1:** **Reach and Grasp components of the DVC theory**.

	Reach	Grasp
1. Musculature	Proximal (upper arm)	Distal (lower arm and hand)
2. Function	Transport hand to target	Shape hand for target purchase
3. Spatial properties	Extrinsic (location and orientation)	Intrinsic (size and shape)
4. Spatial coordinates	Egocentric	Non-Ego and Egocentric
5. Visuomotor channel	Dorsomedial parietofrontal cortex	Dorsolateral parietofrontal cortex

**Figure 1:**
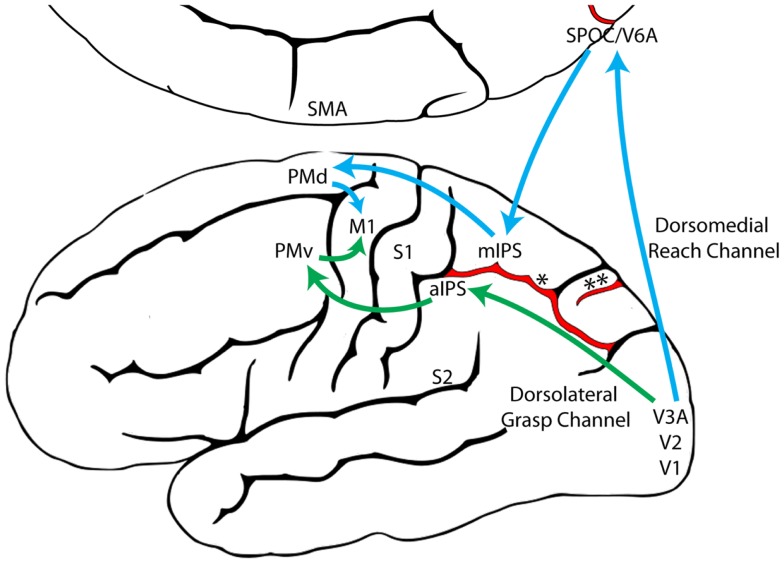
**The dorsomedial Reach pathway (Blue) and the dorsolateral Grasp pathway (Green), adapted from Grafton (31)**. (aIPS, anterior intraparietal sulcus; M1, primary motor cortex; mIPS, medial intraparietal sulcus; PMd, dorsal premotor cortex; PMv, ventral premotor cortex; S1, primary somatosensory cortex; S2, secondary somatosensory cortex; SMA, supplementary motor area; SPOC, superior parieto-occipital cortex; V1, primary visual cortex; V2, secondary visual cortex; V3A, visual area 3A; V6A, visual area 6A; *, intraparietal sulcus; **, parieto-occipital sulcus).

The Reach and the Grasp are subserved by largely segregated visuomotor pathways in occipitoparietofrontal cortex (Figure 1). The dorsomedial Reach pathway projects through the superior parietal lobule via the parietal reach region (PRR), which includes the superior parieto-occipital cortex (SPOC/V6A), medial intraparietal sulcus (mIPS), and anterior precuneous (aPCu). It then projects to dorsal premotor cortex (PMd), and finally to primary motor cortex (M1). The dorsolateral Grasp pathway projects through the anterior intraparietal sulcus (aIPS) to ventral premotor cortex (PMv) and from there to M1 (13, 14, 24–27). Long-train intracortical microstimulation of the dorsomedial pathway elicits reaching movements in awake and anesthetized monkeys, whereas microstimulation of the dorsolateral pathway elicits grasping and/or manipulatory movements (28–30).

**Figure 2:**
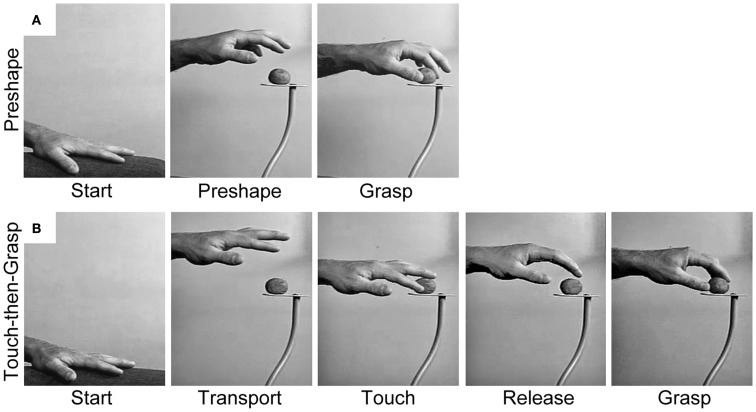
**Representative still frames illustrating (A) the Preshape strategy used to acquire a visible target and (B) the Touch-then-Grasp strategy used to acquire an unseen and unknown or uncertain target**. Note: for the Preshape strategy the Reach and the Grasp are temporally integrated such that the hand preshapes and orients to the intrinsic properties of the target before touching it. For the Touch-then-Grasp strategy the Reach and the Grasp are temporally dissociated such that the hand does not shape to the intrinsic properties of the target until after touching it.

The Dual Visuomotor Channel Theory posits that concurrent visual inputs to the dorsomedial and dorsolateral pathways allow the Reach and the Grasp to be simultaneously executed as a single integrated act (Figure 2A). The preeminent role of vision is illustrated by the act of foviating the target from movement onset until target contact (32). This visual attention is essential for identifying the terminal point of the Reach, i.e., contact locations on the target, and also for coordinating closure of the hand on approach to the target. Nevertheless, non-visual and cognitive inputs may act through the visuomotor Reach and Grasp pathways in order to acquire targets in the absence of visual guidance (33), to produce pantomime Reach and Grasp movements (34, 35), and also to produce spontaneous Reach and Grasp gestures associated with speech (36).

## Visual Occlusion Dissociates the Reach and the Grasp in Healthy Adults

One approach to dissociating the Reach and the Grasp is to manipulate the relative extrinsic or intrinsic properties of a single visual target, but this manipulation has produced ambiguous results. For example, Jeannerod (10) finds that when the size of a visible target is changed unexpectedly, the Grasp is altered but the Reach is not. In contrast, Jakobson and Goodale (37) find that both the Reach and the Grasp are altered. The difficulty in dissociating the Reach and the Grasp with this approach is that when the shape or location of the visual target is changed, both extrinsic and intrinsic target properties are altered resulting in concurrent adjustments in both the Reach and the Grasp.

An alternative way to dissociate the Reach and the Grasp is to remove vision, such that the extrinsic and intrinsic properties of the target must be determined non-visually. Karl et al. (38) asked blindfolded participants to reach for targets of varying size: a blueberry, donut ball, and orange slice. Targets were randomly presented, one at a time, on a pedestal in front of the participants so that they would not know which target they were reaching for on any given trial. In performing the task, participants advanced an open hand above and then down onto the target, often palpitating in the region of the target until touching it. The dorsal trajectory and open digits appeared to enhance the chances of target contact. After touching the target, the participants used haptic cues to shape the digits for grasping. Sometimes the hand released contact with the target before the digits preshaped and closed to Grasp. At other times, the target was stabilized or manipulated by some digits while the remaining digits shaped to Grasp. Hand scaling after target contact was equal to that of visually guided hand preshaping. Thus, when the extrinsic and intrinsic properties of the target cannot be visually determined, the prehensile act decomposes into sequential Reach and Grasp movements, each guided by somatosensation. The Reach, likely mediated by proprioception, is performed first and serves to locate the target by touching it. Only after contact do the hand and digits shape to haptic cues in order to Grasp the target. This two-staged act is termed a Touch-then-Grasp strategy and is illustrated in Figure 2B.

A variation of this experiment had participants learn about the extrinsic and intrinsic properties of the target through repeated non-visual experience (39). Blindfolded participants reached 50 times for a donut ball. Although initially unknown, both the location and size of the target could be learned through repetition. As was found in the unknown target experiment, participants persisted in using a dorsal Reach trajectory, in which the hand approached the target from above and an open hand and digits were used to locate the target by touching it. Nevertheless, within a few trials the participants began to preshape hand aperture to the size of the target before touching it. Scaling of hand aperture became indistinguishable from that of sighted participants. Thus, previous non-visual experience had differential effects on the Reach and the Grasp such that a dorsal Reach trajectory became coupled with a preshaped Grasp. Another experimental variation had participants perform the task using peripheral vision, a manipulation that provided enough visual information to identify each target while still degrading information about target size and location (Hall et al., unpublished). Similar results were obtained, participants maintained a dorsal Reach trajectory but could scale hand aperture to target size before touching it albeit, less accurately than under foveal vision.

The finding that previous somatosensory experience can instruct accurate hand preshaping for the Grasp raises the question of whether online haptic inputs could produce fully integrated Reach and Grasp movements similar to that of visually guided prehension. Online haptic feedback is known to be available in acts such as reaching for a part of the body or objects on the body. Thus, participants were asked to reach for one of three different sized food targets that were randomly placed in their mouth by the experimenter (40). When reaching to grasp the target, participants preshaped and oriented the hand prior to target contact, closed the digits in anticipation of target contact, and successfully grasped the target on the first attempt. Scaling of hand aperture was as accurate, and for some food items, more accurate, than that of visually guided grasping. Thus, online haptic information from a target held in the mouth is as informative as online vision for guiding integrated Reach and Grasp movements.

The behaviors called upon in these studies resemble many everyday actions in which people reach for and manipulate objects under degraded visual conditions. Such acts include reaching for objects in the dark, reaching for objects contacting the body (41, 42), or sequential reaching acts in which one object is grasped while visual attention is directed to a subsequent target. Collectively, these studies support the idea that somatosensation and vision both have access to the Reach and Grasp pathways (43–47). As will be discussed below, this conclusion further suggests that somatosensation may have been formative in the evolution of distinct Reach and Grasp movements and their underlying neural substrates.

## Visual Impairment Dissociates the Reach and the Grasp after Brain Injury

The Reach and the Grasp are also dissociated after localized brain injury that disrupts visual input to one or both of the visuomotor pathways. Patients with such injury display optic ataxia; an impairment in visually guided hand movements despite normal visual perception (48, 49). Recent work with optic ataxic patients support the postulate of the Dual Visuomotor Channel Theory that the visuomotor pathways of the Reach and the Grasp are subject to a double dissociation.

A number of patients with damaged visual inputs to the Grasp, but not the Reach, pathway have been described (50, 51). These patients have no problem reaching to the location of a visual target and consistently touch it on the first attempt; however, they use an open hand to do so and only close their digits to grasp the target after touching it. Thus, these patients seemingly adopt a modified Touch-then-Grasp strategy. They use vision to determine the target’s extrinsic properties (location) but are unable to use vision to determine the target’s intrinsic properties (size and shape) and thus cannot preshape the hand to Grasp prior to target contact. Instead they rely on haptic cues after target contact to shape their digits to the contours of the target in order to Grasp it.

Cavina-Pratesi and colleagues (52) describe the reverse condition, in which a patient cannot perform a visually guided Reach but can perform a visually guided Grasp. The patient, M.H., suffered an anoxic episode, disrupting visual inputs to the Reach but not the Grasp pathway. M.H. accurately opens, preshapes, and closes his hand to Grasp a visual target, but only if the target is located adjacent to his hand; i.e., if he doesn’t have to Reach for it. If he does have to Reach for it, he must first locate it by touch before shaping his hand to Grasp it: “Presumably M.H., wittingly or unwittingly, compensates for the direction and distance errors resulting from his damaged visual reaching network, by habitually opening his hand widely: the wider the hand aperture, the higher the probability of successfully acquiring the object.” M.H.’s visually guided Reach movements are inaccurate regardless of whether the movement is directed inward (toward his body) or outward (away from his body), indicating that his deficit is related to visual guidance of the Reach and not the location of the target within egocentric space. Thus, M.H. can use vision to guide his hand in relation to the intrinsic (size and shape) but not extrinsic (location) properties of a target.

**Figure 3:**
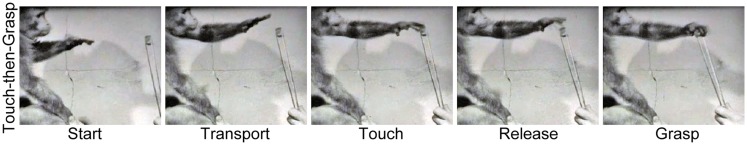
**Representative still frames illustrating the Touch-then-Grasp strategy used by a macaque monkey to acquire a visible target 5 months after a bilateral corticospinal tract lesion**. Note: even though vision is available the monkey advances an open hand toward the target, and only shapes and closes the hand to grasp the target after it has been touched [videos provided by Lawrence (54)].

The neural substrates that integrate the Reach and the Grasp under visual control may extend beyond the cortex into the spinal cord. Karl and Whishaw (53) re-examined the Reach and the Grasp movements of monkeys with bilateral corticospinal tract (CST) lesions, first described by Lawrence and Kuypers (54). The analysis suggests that these monkeys may also use a Touch-then-Grasp strategy to acquire visual targets. They Reach toward the target using an open and extended hand and often miss the target on the first attempt. They then palpitate the hand in the vicinity of the target until they touch it. After initial contact, the hand releases contact with the target, re-shapes, re-orients, and finally closes to Grasp the target (Figure 3). Similar impairments in hand preshaping have also been reported following more selective CST lesions in monkeys (55–57).

Taken together these lesion studies suggest that the visuomotor pathways of the Reach and the Grasp are separate. They also suggest that if brain injury deprives a subject of visual information, somatosensory mediated Reach and Grasp movements are adopted. Finally, it is possible that direct corticomotoneurons in primates mediate the motor output for visual control of the Reach and the Grasp pathways. It is instructive that direct corticomotoneurons and the dorsal visual stream evolved concurrently in the primate lineage.

## Dissociation of the Reach and the Grasp in Early Infancy

At about 5 months of age human infants begin to haphazardly reach for visual targets, gradually becoming more accurate at bringing a single hand to the target, and finally developing precision grips to grasp (58–63). We have re-examined the development of infant reaching in order to determine whether the Reach and the Grasp have different developmental profiles. The results show that the Reach and the Grasp emerge independently as PreReach and PreGrasp movements in early development and require a significant length of time to become fully integrated under visual control.

Young infants produce a variety of PreReach movements before they can direct a single hand to the location of a visual target. From birth infants can orient the eyes and head to a visual target (64, 65). Soon after, they reach for the target with the mouth by thrusting the head forward and flexing the abdominals [Ref. (66); Video S1 in Supplementary Material], eventually they use a fisted hand to swipe and wave at the target (67). Consummation of these PreReach movements into a targeted, visually guided Reach only emerges at about 5 months of age. Initially an open hand advances along a jerky trajectory to make imprecise contact with the target (61). This ability develops equally whether the infant has sight of their hand or not and successful contact with the target is signaled by haptic rather than visual feedback (68, 69). However, by 7–9 months, visual control of the Reach improves significantly such that the location and orientation of the open hand accurately reflect the extrinsic properties of the target at the moment of target contact (53, 70, 71).

Young infants also produce a variety of PreGrasp movements before they can preshape the hand and digits to match the contours of a visual target. At birth the digits display a closed and flexed posture, but by 1 month they adopt a collected posture in which the hand is relaxed and partially open (72). Nevertheless, newborn infants will close the digits on an object that makes haptic contact with the palm (73) and by at least 4 months of age infants can use haptic cues to shape the hand to match the contours of an object (74). By 2 months of age infants start “hand babbling,” producing a variety of spontaneous but complex digit movements that form a variety of Grasp configurations. Movements include extension and flexion of individual digits, sequential digit movements, and pressing individual digit pads together to form vacuous pincer and precision grips [Ref. (75); Video S2 in Supplementary Material]. At 4 months, these movements become self-directed and are used to grasp the infant’s own body or clothing. In performing these movements, infants do not look at their hands, suggesting that the movements are shaped by somatosensation rather than by vision.

**Figure 4:**
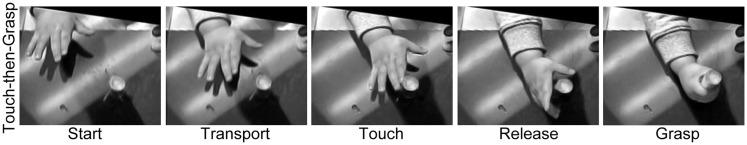
**Representative still frames illustrating the Touch-then-Grasp strategy used by a 7-month old human infant in order to acquire a visible target**. Note: even though vision is available the infant advances an open hand toward the target, touches the target, and, only after contact, then re-orients, shapes, and closes, the hand to Grasp it.

Not only do the Reach and the Grasp emerge independently in early development, but they require a long developmental period to be integrated under online visual control. When infants first start to Reach to visual targets, they advance an open hand along a jerky trajectory, often missing the target on the first attempt or making multiple contacts between the open hand and target before closing to Grasp it. Thus, they do not preshape the hand to the target and use a Touch-then-Grasp strategy similar to that described above for unsighted adults. As infants age, they become more accurate at using vision to direct an open handed Reach to the target on the first attempt; however, they do not preshape the hand and haptic contact with the target continues to instruct shaping of the Grasp, similar to the first set of optic ataxic patients described in the previous section [Figure 4; Ref. (68, 76)]. Thus, the Reach and the Grasp are dissociated in early development and complete integration of the two movements under visual control, such that the hand accurately preshapes prior to target contact, does not appear to be complete until at least 2 years of age (53, 77).

In summary, analyses on the development of prehension provide evidence that the Reach and the Grasp follow independent developmental profiles. Initially, both the Reach and the Grasp emerge under somatosensation and only later come under visual control. Even then, visual guidance of the Reach develops before visual guidance of the Grasp. Finally, integration of the Reach and the Grasp under visual control only appears after a protracted developmental time course lasting into early childhood.

## Distinct Evolutionary Origins for the Reach and the Grasp

The preceding lines of evidence show that somatosensory and visual information have equal access to the neural pathways that control the Reach and the Grasp. When vision is removed or limited, as occurs with visual occlusion, brain injury, or early in development, the Reach and the Grasp are dissociated by a Touch-then-Grasp strategy that maximizes the use of haptic feedback for guiding each movement independently. Nevertheless, the Reach and the Grasp can be integrated under non-visual control, similar to visually guided prehension, if online haptic feedback concerning the target is available. In the following section we will consider evidence that haptically mediated Reach and Grasp movements are phylogenetically older than those guided by vision.

In phylogenetically early quadrupeds, the neural control of the forelimbs and hindlimbs is tightly coupled to subserve locomotion, but even when stepping a forelimb has independence. Forelimb stepping is achieved by first flexing the forelimb to release contact with the substrate and then extending it to re-establish contact at another location (78). Semi-independent control of a single forelimb likely evolved to allow animals to circumvent obstacles and to navigate over uneven terrain (5, 79–81). Complete independence of a single forelimb allowed the stepping movement to be adapted for a variety of non-locomotor functions such as pushing, swatting, or digging. For instance, a polar bear may flex and extend a single forelimb in order to pin a slippery fish to the ground, a cat may flex and extend a single forelimb to swat at a fly, or a boar may flex and extend a single forelimb to uncover a food item covered by soil. Thus, the wide range of independent forelimb movements produced by various animals, including Reach movements, may be derived from a common origin, stepping.

**Figure 5:**
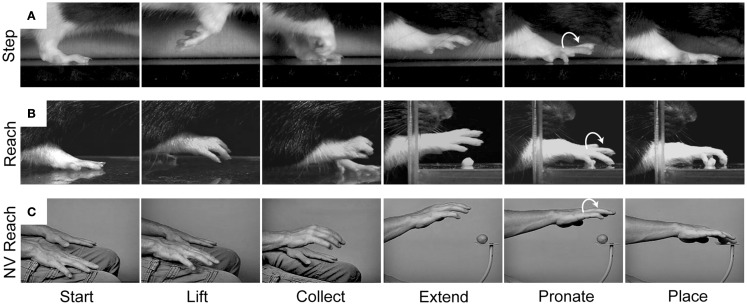
**Representative still frames illustrating the kinematic structure of (A) a rat forelimb stepping movement, (B) a rat Reach movement, and (C) a human non-visual Reach movement**. Note: all three movements share a common kinematic structure in which the hand is first *lifted* from the underlying substrate, the digits *collect* and *extend* as the arm is advanced forward, and the hand *pronates* before being *placed* on the new substrate. Adapted from Whishaw et al. (83) and Karl et al. (38).

Our behavioral and kinematic analyses reveal similarities between forelimb stepping and the Reach movement which support the idea of common origin (Figure 5). We have examined a variety of movements in rodents and primates, including walking in rats, crawling in humans, and climbing and reaching in both species. In all of these behaviors, the forelimb movement is initiated by flexing the elbow and *lifting* the hand from the substrate. The digits then flex and close in a *collected* posture as the limb is transported forward. The digits then open and *extend* as they approach the target. The hand then *pronates* in the lateral to medial direction and is finally *placed* on the target or substrate (38, 82, 83). Thus, a number of kinematic similarities shape both forelimb stepping and the Reach movement.

For movements of stepping and its derivatives, vision is not essential. Vision is usually directed ahead of the limb’s target (84, 85). Thus, the step is performed in the absence of online sensory control until it receives haptic confirmation associated with limb placement. A rat may use vibrissae cues to signal where to step (83) but in its forward movement, this sensory signal precedes the step. Likewise, a rat may use olfactory cues to locate a food item that it will retrieve with a Reach, but the animal must displace its head in order to clear a path for the hand to the target (86). As a result, rats perform both stepping and Reach movements in the absence of online visual control (83, 87). Thus, like a blindfolded human reaching for an unknown target, the rat does not preshape the hand prior to target contact and cannot learn to do so even with extended training (88). Detailed information on the sensory control of the forelimb for most actions in most animal species is not available, but available evidence suggests that visual guidance is not prominent in species other than primates. Taken together, comparative evidence for kinematic similarities in the structure of forelimb transport, collection, and lateral to medial pronation, coupled with the distinct absence of hand preshaping, argues that only the Reach movement, not an integrated Reach-to-Grasp movement, is derived from forelimb stepping.

The Grasp action, especially grasping a food item, is a common forelimb movement in many vertebrate orders (16, 17, 89). Grasping not only involves holding a food item and bringing it to the mouth with a hand, but taking an item from the mouth with a hand or taking it from one hand with the other hand, as well as manipulating the item in preparation for consumption. Furthermore, in various non-primate species, specialized hand and digit movements may be used to Grasp and remove the hard shell from a sunflower seed, the spiky legs from a cricket, or the fleshy peel from an orange (90–93). In all of its manifestations these Grasp movements are guided by hapsis. Thus, the demands of a diverse diet have led to the evolution of dexterous and haptically sensitive hands (94–96).

**Figure 6:**
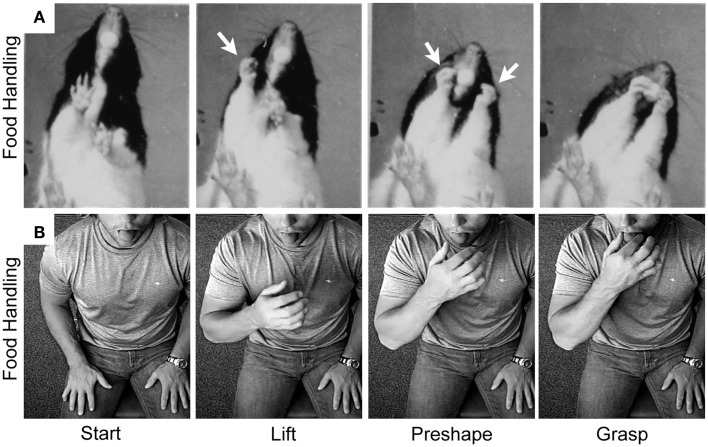
**Representative still frames illustrating the kinematic structure of (A) a rat food handling movement and (B) a human food handling movement**. Note: both movements share a common kinematic structure in which the hand is first *lifted* from the substrate, the digits then *preshape* to the target, and finally the digits close on approach to the target in order to *grasp* it. White arrows indicate hand preshaping in the rat. Adapted from Whishaw et al. (97) and Karl et al. (38, 40).

**Figure 7:**
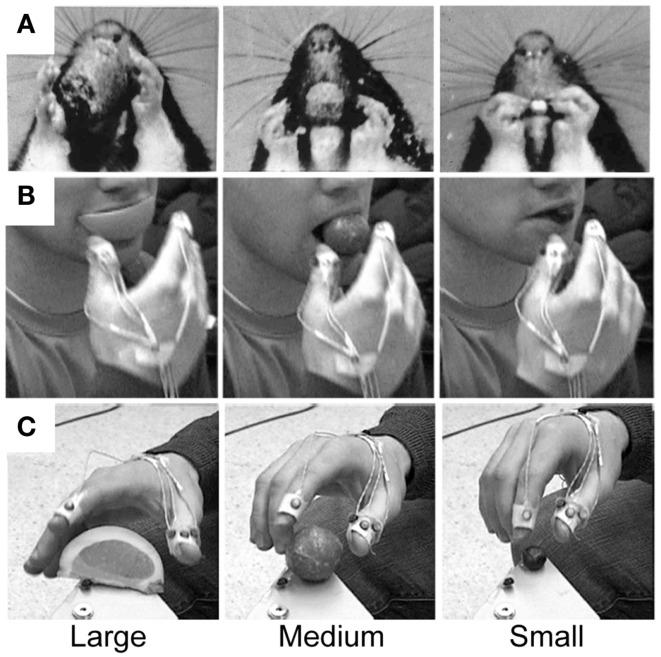
**Representative still frames illustrating hand preshaping before touching the target in (A) rat food handling movements, (B) human food handling movements, and (C) human visually guided Grasp movements**. Note: in all three situations online haptic (food handling) or visual (Grasp) information is available to guide hand preshaping such that a large peak hand aperture is used to Grasp a large food item, an intermediate peak hand aperture is used to Grasp a medium-sized food item, and a small peak hand aperture is used to Grasp a small food item. Adapted from Whishaw et al. (97) and Karl et al. (40).

The many manipulations made by the hand in handling food require preshaping by both the hand and the mouth to receive the food item (97). These preshaping movements are the likely origin of hand preshaping for the primate Grasp. Comparisons of rat and human hand preshaping prior to retrieving a food item from the mouth are illustrated in Figures 6 and 7. The human is blindfolded and the location of the rodent’s eyes prevent it from observing its hands. For both species, online haptic feedback from the food in the mouth guides hand preshaping in order to Grasp the food item. The movement is initiated by *lifting* the hand from a substrate, *preshaping* the hand to the size of the target, and closing the digits on approach to the target in order to *Grasp* it (Figure 6). Even though rodents are unable to preshape the hand when reaching to a distal target; they, like primates, readily use oral hapsis to scale hand aperture to the size of a target in the mouth (Figure 7), which is also similar to visually guided hand preshaping displayed by primates (40, 97).

**Figure 8:**
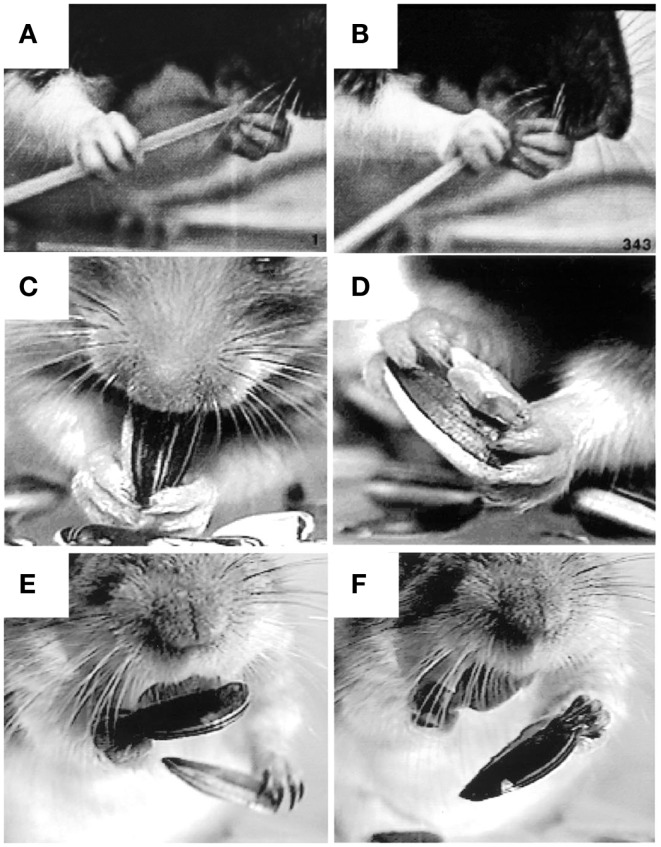
**Representative still frames illustrating specialized grip configurations and independent digit movements in rodents during food handling**. **(A,B)** A rat eating uncooked spaghetti, the left hand holds the pasta near the mouth with the digit tips (a modified precision grip) while the right hand uses a scissor grip between digits 4 and 5 to push the pasta toward the mouth. **(C,D)** A hamster eating a sunflower seed, both hands hold the seed in a modified precision grip between digit 1 (the thumb) and digits 2 and 3 as the mouth bites into the shell. Two objects can also be held at once, the seed is held in a modified (bilateral) pincer grip between digits 1 and 2, the shell is held in a bilateral power grip between the palm and digits 3 and 4, while digit 5 is positioned on the ventral surface of the seed, likely to stabilize the grip on both objects. **(E,F)** A Mongolian gerbil eating a sunflower seed. A bite from the incisors is used to open the shell (not shown). The bottom half of the shell is held in the digit tips as the left hand uses a precision grip to grasp and discard the top portion of the shell. The left hand then grasps the bottom half of the shell in the digit tips (precision grip) and discards it as the right hand uses a precision grip to hold the seed in the mouth. Adapted from Whishaw et al. (93), Whishaw et al. (92).

After the target is grasped a large and varied vocabulary of specialized grip configurations and independent digit movements may be used to manipulate, explore, or stabilize the food item (Figure 8). For further descriptions in non-primates see (17, 91–93, 97). For descriptions in primates see (98–100). That both rodents and primates use haptic information to Grasp a food item in the mouth suggests that haptically guided hand preshaping, as well as manipulatory digit movements, predate visually guided Grasp movements in primates (40, 96).

## Distinct Anatomical Origins for the Reach and the Grasp

In addition to classic work (5), recent electrophysiological and brain imaging studies in non-human primates and humans support the notion that cortical control of the Reach could be derived from a pre-existing locomotion pathway in parietofrontal cortex. Like the Reach, stepping appears to be mediated by a dorsomedial pathway in parietofrontal cortex. Electrical stimulation of this pathway elicits bilateral movements of the forelimbs and hindlimbs that resemble spontaneous running or leaping in monkeys (28–30, 101). In humans, regions of dorsomedial posterior parietal cortex (PPC) mediate reaching to visible targets with the arms and hands, but also subserve pointing and stepping movements to visible targets with the foot (102–106). Although the stepping and Reach pathways overlap in dorsomedial PPC, they appear to diverge in frontal cortex. Thus, regions of overlap (SPOC/V6A, mIPS) may code for a specific behavioral function; i.e., transport of a limb to a different spatial location, whereas regions of divergence (PMd/SMA and M1) might specify the body part used to execute that behavior, i.e., the foot (stepping) or the hand [forelimb stepping/Reach; Ref. (104)].

Food handling, like grasping, may be mediated by a dorsolateral pathway in parietofrontal cortex. Electrical stimulation of this dorsolateral pathway elicits hand-to-mouth movements, in which the hand is lifted toward an open mouth and the digits shape to Grasp (28–30, 101). In humans, a similar region in the inferior parietal lobule is activated when performing grasping movements with either the mouth or hands (107). Furthermore, aIPS, the parietal region of the dorsolateral Grasp pathway, receives strong somatosensory inputs (29, 108–110) and mediates grasping and manipulatory digit movements directed toward both visual and haptic targets (44–46, 111, 112). Thus, it is possible that, like the stepping and Reach pathways, the food handling and Grasp pathways may overlap in parietal cortex (aIPS), which might code for a specific behavioral function, i.e., shaping a body part to grasp/manipulate a target, whereas regions of divergence (M1) could specify the body part used to execute that behavior, i.e., the mouth (bite) or the hand (Grasp).

Interestingly, lesions to V6A, a crucial node in the dorsomedial Reach pathway, disrupt both Reach and Grasp movements (113), although, as demonstrated by Cavina-Pratesi et al. (52), the Grasp impairments could emerge as a secondary consequence of misreaching. Nevertheless, V6A receives inputs from AIP [the macaque homolog of human aIPS; Ref. (108, 114)] and contains orientation- and grip-selective neurons (115–117). Thus, V6A could have originally evolved to serve the Reach, but through its connections with AIP, it may also monitor preshaping of the Grasp as the hand is advanced toward the target. Thus, primate V6A may serve as a visuoproprioceptive “integrator,” ensuring that visually guided Reach and Grasp movements unfold in temporal synchrony (116). Indeed, the neural substrate that integrates the Reach and the Grasp must emerge early in the visuomotor pathways in order to integrate the two movements from action onset. One way to determine whether the grip-selective properties of neurons in V6A are intrinsic to this cortical area, or emerge in response to inputs from AIP, would be to selectively lesion AIP while observing the effect on grip-selective neurons in V6A.

Although non-primate species do not display visually guided hand preshaping during reaching, behavioral evidence suggests that the sensorimotor representations of the Reach and the Grasp should be similar to that of primates with respect to motor control. For instance, the rat has a well-developed forelimb representation in anterior motor cortex consisting of a relatively smaller rostral forelimb area (RFA) and a larger caudal forelimb area [CFA; for a reviews see Ref. (118, 119)]. Microelectrical stimulation of these regions produces brief movements of distal and proximal regions of the contralateral forelimb, respectively. Longer train electrical stimulation in the RFA is more likely to elicit movements involving the hands, including grasping, whereas stimulation in the CFA elicits whole limb movements (120), some of which resemble reaching. Inactivation of these regions disrupts Grasp and Reach movements respectively (Brown and Teskey, unpublished). Additionally, results from brainstem stimulation in freely moving rats suggest separate subcortical regions mediate the Reach (stepping movements) and the Grasp (food handling movements). For example, forced forelimb movements are obtained by electrical stimulation in the region of nucleus gigantocellularis whereas fictive eating (the rats sits on its haunches and engages in food handling and eating without food) from the region of the locus coerulelus (121).

We also suggest that descending projections from cortical motor regions may form the efferent control of the cortical visuomotor Reach and Grasp pathways. The direct projections of the CST are distinctive in primates (122) but have been associated with the production of independent digit movements (54, 123, 124). Yet there are many difficulties with the independent digit theory, including definitional difficulties related to independent digit movements as well as evidence that deficits following cortical injury are related to movement synergies, not independent digit control (125, 126). Independent digit movements are also distinctive in the hand babbling movements of infants as young as 2 months of age (75), well before maturation of the direct connections of the CST is complete (127, 128). In the earliest stages of development and following CST lesions in primates, prehension resembles optic ataxia in that the Reach and the Grasp do not appear to integrate under visual control, but are characterized instead by a distinct absence of hand preshaping as well as the use of modified Touch-then-Grasp strategies. The prolonged developmental period required to integrate the Reach and the Grasp also seems to parallel the long maturational period characteristic of the direct projections of the CST. Taken together, this evidence seems to suggest that, in primates, visual integration of the Reach and the Grasp co-evolved with direct corticospinal projections from motor cortex.

Collectively, anatomical studies confirm predictions from behavioral work that separate pathways should subserve the Reach and the Grasp in non-primate species and that these species could be further examined to identify the neural origins of primate Reach and Grasp movements. Specifically, it is proposed that the neural circuits for stepping are the evolutionary antecedent for the Reach whereas the neural circuits for food handling are the evolutionary antecedent for the Grasp. Early in their evolution these movements were importantly dependent on non-visual guidance, including somatosensation and olfaction, whereas visual control of the Reach and the Grasp appears to have emerged later as a primate specialization. The proposition that visually guided Reach and Grasp movements might be derived from pre-existing non-visual stepping and food handling circuits fits well with recent evidence that movement representations in primate parietofrontal cortex are both effector- and modality-independent (42).

## Conclusion

Healthy adults use vision to integrate the Reach and the Grasp into a unified prehensile act by preshaping the hand and digits to the size and shape of a visible target as the hand is advanced toward it. This behavior is critically dependent on foveal vision. Nevertheless, when visual inputs are limited or disrupted as occurs during early development, under visual occlusion, or following brain injury, prehension decomposes into its constituent movements: a Reach that advances an open hand in order to haptically locate the target and a haptically guided Grasp that shapes the hand and digits for target purchase.

**Figure 9:**
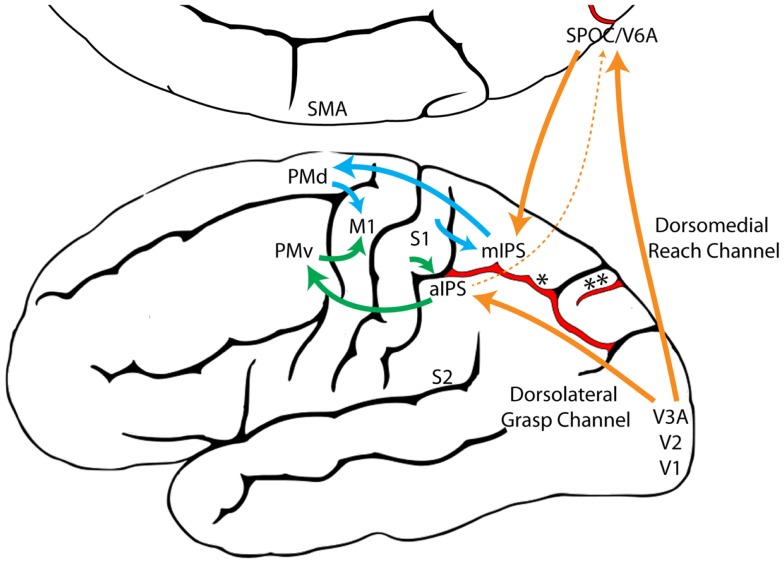
**A model illustrating the proposed evolutionary origins for dual visuomotor Reach and Grasp channels in primate parietofrontal cortex**. The original dorsomedial stepping/Reach circuit (blue) and the dorsolateral food handling/Grasp circuit (Green) evolved first and were subsequently harnessed by the primate visual system (Orange) through neural re-use (129). (aIPS, anterior intraparietal sulcus; M1, primary motor cortex; mIPS, medial intraparietal sulcus; PMd, dorsal premotor cortex; PMv, ventral premotor cortex; S1, primary somatosensory cortex; S2, secondary somatosensory cortex; SMA, supplementary motor area; SPOC, Superior parieto-occipital cortex; V1, primary visual cortex; V2, secondary visual cortex; V3A, visual area 3A; V6A, visual area 6A, *, intraparietal sulcus, **, parieto-occipital sulcus).

The independence of the Reach and the Grasp under non-visual control supports the proposition of the Dual Visuomotor Channel Theory that the neural substrates of the Reach and the Grasp are distinct and derived from different evolutionary origins. Collective evidence suggests that the primate Reach is one of a number of species-specific adaptations derived from forelimb stepping, whereas the primate Grasp is one of a number of species-specific adaptations derived from food handling. Thus, distinct motor circuits for the “Reach” and the “Grasp” may have emerged relatively early in evolution and were likely influenced more by non-visual than visual inputs. Expansion of the primate visual system would have given rise to a number of new connections between occipital and parietofrontal cortex, allowing vision to harness these pre-existing “Reach” and “Grasp” circuits resulting in multiple visuomotor pathways from occipital to parietofrontal cortex (Figure 9). No longer constrained by the necessity of haptic control, the Reach and the Grasp could be executed simultaneously, rather than sequentially, giving primates the unique ability to preshape the hand to the intrinsic properties of a visual target before touching it.

Finally, distinct neural and evolutionary origins for the Reach and the Grasp would allow for a multiplicity of Grasp movements including various single handed pincer, precision, or power grasps, as well as any combination of two handed grasps to be combined with a multiplicity of Reach movements including single handed reaches, two handed reaches, pushes, throws, or swats, all of which can executed under various forms of sensory or cognitive control. Thus, the proposition that distinct motor circuits for the Reach and the Grasp evolved separately and only came under visual control late in the evolutionary process supports the idea that the Reach and the Grasp pathways in parietofrontal cortex are accessed not only by vision, but also by a variety of non-visual and cognitive inputs in order to produce a diverse repertoire of adaptive behaviors upon which natural selection may act.

## Conflict of Interest Statement

The authors declare that the research was conducted in the absence of any commercial or financial relationships that could be construed as a potential conflict of interest.

## Supplementary Material

The Supplementary Material for this article can be found online at http://www.frontiersin.org/Journal/10.3389/fneur.2013.00208/abstract

Video S1 **PreReach movements made with the mouth in a 2-month old human infant**. Adapted from Foroud and Whishaw (66).

Video S2 **Hand babbling in a 2-month old human infant**. Note the production of independent digits movements and vacuous pincer and precision grips. Adapted from Wallace and Whishaw (75).
